# circ-CFH promotes the development of HCC by regulating cell proliferation, apoptosis, migration, invasion, and glycolysis through the miR-377-3p/RNF38 axis

**DOI:** 10.1515/biol-2022-0029

**Published:** 2022-03-23

**Authors:** Zengyin Chen, Juan Du, Chen Yang, Guangju Si, Yuxin Chen

**Affiliations:** Department of Hepatobiliary Surgery, Chengyang District People’s Hospital, Qingdao, 266000, Shandong, China; Department of Hepatobiliary Surgery, Qingdao People’s Hospital, No. 600 Changcheng Road, Chengyang District, Qingdao, 266000, Shandong, China

**Keywords:** circRNA, circ-CFH, miR-377-3p, RNF38, HCC

## Abstract

Circular RNAs (circRNAs) have previously been confirmed to function as vital regulators in multiple human cancers, including hepatocellular carcinoma (HCC). This study aimed to clarify the role and underlying molecular mechanisms of circ-CFH in HCC. circ-CFH was overexpressed in HCC tissues and cells, and the downregulation of circ-CFH inhibited the development of HCC by repressing cell proliferation, migration, invasion, and glycolysis while enhancing apoptosis *in vitro*, as well as inhibited tumor growth *in vivo*. miR-377-3p was negatively regulated by circ-CFH, and silencing of miR-377-3p abolished circ-CFH knockdown-mediated effects on HCC cells. Moreover, overexpression of miR-377-3p could impede the HCC process by targeting RNF38. Mechanistically, the circ-CFH/miR-377-3p/RNF38 axis regulated the progression of HCC cells, which might provide new diagnostic markers for HCC.

## 1 Introduction

Hepatocellular carcinoma (HCC) is a major pathological type of primary liver cancer, accounting for 75–85% of cases [1]. The infection of hepatitis B/C virus, alcoholism, smoking, obesity, and aflatoxin-contaminated foodstuffs are the main incentives for HCC [2]. The poor prognosis of HCC patients is commonly associated with the high frequency of metastasis and recurrence, as well as the poor response to treatment [3,4]. It is urgently needed to investigate cellular and molecular pathways involved in HCC pathogenesis to find new targets for HCC management.

Circular RNAs (circRNAs) are a group of closed-loop structure RNAs with evolutionary conservation and cell/tissue type-specific expression [5]. CircRNAs participate in gene expression regulation by acting as miRNA sponges, which is a common regulatory pattern in cancer [6]. Recently, there has been increasing evidence that circRNAs work as efficient regulators in the development of tumors [7,8]. circ-CFH (circ_0015756) is derived from the complement factor H (CFH) gene and locates on chr1 (196706604-196712758), which has been suggested to function as a tumor promoter in ovarian cancer, glioma, and hepatocellular carcinoma [9,10,11]. Liu et al. uncovered that circ-CFH could promote proliferation, invasion, and migration in HCC [12]. Guo et al. also confirmed that circ-CFH enhanced the development of HCC *in vitro* and *in vivo* [10]. However, the mechanism underlying circ-CFH in HCC is insufficient and still needs subsequent analysis.

MicroRNAs (miRNAs) could mediate biological behaviors such as proliferation, differentiation, apoptosis, and metastasis via regulating gene expression at the post-transcriptional level by targeting the 3′untranslational region (3′UTR) of target mRNAs [13]. For example, Chen et al. confirmed that miR-379-5p repressed the development of HCC by targeting focal adhesion kinase (FAK) [14]. miR-377-3p is a well-explored miRNA, which was confirmed to act as a tumor suppressor in several types of tumors, including triple-negative breast cancer [15], colorectal cancer [16], and glioma [17]. In addition, Zhan et al. disclosed that miR-377-3p was regulated by hsa_circ_103809 and participated in the regulation of HCC progression [18]. Liu et al. also found that miR-377-3p was sponged by circVAPA and inhibited the proliferation of HCC [19]. Hence, miR-377-3p might be a potential therapeutic target for HCC, whereas its regulatory mechanism in HCC needs further investigation.

RING finger protein 38 (RNF38) is widely expressed in human tissues and belongs to the RNF protein family that plays significant roles in the physiology and pathologies of human cancers [20,21]. For instance, RNF38 could mediate epithelial-to-mesenchymal transition (EMT)-related molecules expression, thereby regulating proliferation and metastatic capacity of lung cancer cells [22]. In HCC, it has been reported that RNF38 overexpression promoted cell migration, invasion, and epithelial–mesenchymal transition but inhibited apoptosis in HCC by facilitating transforming growth factor-β (TGF-β) signaling [23]. However, the regulatory mechanism of RNF38 in HCC progression needs further elucidation.

Here, the expression levels of circ-CFH, miR-377-3p, and RNF38 were investigated in HCC, and the possible association among circ-CFH, miR-377-3p, and RNF38 was confirmed in HCC cells. We confirmed that overexpression of circ-CFH facilitated HCC progression by the miR-377-3p/RNF38 axis, which may serve as a potential therapeutic target for HCC.

## 2 Materials and methods

### 2.1 Patient specimens

HCC tissues (*n* = 30) and paired nontumorous tissues (*n* = 30) were collected from HCC patients at Qilu Hospital, Shandong University. Briefly, the tumor tissues were promptly frozen in liquid nitrogen and then stored in a −80°C refrigerator. Moreover, the written informed consents were acquired from patients or their guardians before the surgical procedure. The clinical–pathological features of 30 HCC patients are shown in Table 1.

**Table 1:** Correlation between circ_CFH expression and the clinical–pathological features of 30 HCC patients

Characteristic	All cases	circ_CFH expression		*P*-value
		High (*n* = 15)	Low (*n* = 15)	
Gender				0.713
Male	17	9	8	
Female	13	6	7	
Age (years)				0.143
<60	16	6	10	
≥60	14	9	5	
Tumor size (cm)				0.456
≥5	18	10	8	
<5	12	5	7	
Tumor metastasis				0.028*
Absent	14	4	10	
Present	16	11	5	
Microvascular invasion				0.464
Absent	14	6	8	
Present	16	9	7	
TNM stages				0.028*
I/II	16	5	11	
III/IV	14	10	4	

*P < 0.05.


**Informed consent:** Informed consent has been obtained from all individuals included in this study.
**Ethical approval:** The research related to human use has been complied with all relevant national regulations, institutional policies and is in accordance with the tenets of the Helsinki Declaration, and has been approved by the Ethics Committee of Qilu Hospital, Shandong University.

### 2.2 Cell lines

HCC cell lines (Huh7 and SNU387) and normal hepatic cell lines (THLE-2) were obtained from the Shanghai Institutes for Biological Sciences (Shanghai, China). These cells were propagated in Dulbecco’s modified Eagle’s medium (Life Technologies, Carlsbad, CA, USA) supplemented with 10% (v/v) fetal bovine serum (FBS; Life Technologies), 100 U/mL penicillin (Life Technologies), and 100 mg/mL streptomycin (Life Technologies) under standard culture conditions (5% CO_2_, 37℃).

### 2.3 Real-time quantitative polymerase chain reaction (RT-qPCR)

Total RNA was isolated using the RNA extraction kit (Invitrogen, Carlsbad, CA, USA) in accordance with the manufacturer’s protocols, and then 500 μg of total RNA was reverse-transcribed into complementary DNA (cDNA) using the PrimeScript™ RT reagent kit (Takara, Dalian, China). For the detection of miR-377-3p expression, the Hairpin-it TM miRNAs qPCR Quantitation Kit (Genepharma, Shanghai, China) was used. The RT-qPCR assay was conducted using SYBR Green Real-Time PCR Master Mix (Applied Biosystems, Carlsbad, CA, USA) on a Thermal Cycler CFX6 System (Bio-Rad, Hercules, CA, USA) and quantified with the 2^−ΔΔCt^ method. The expression levels of circ-CFH and RNF38 were normalized to glyceraldehyde-3-phosphate dehydrogenase (GAPDH), while the expression level of miR-377-3p was normalized to small nuclear RNA U6. The sequences of primers are listed in Table 2.

**Table 2:** The sequences of primers used for RT-qPCR

Names	Primers	Sequences
circ-CFH	Forward	5′-AAATGGAAACTGGACGGAAC-3′
	Reverse	5′-TCCCATGTGCATAACTTTCTTG-3′
miR-377-3p	Forward	5′-GCCGAGATCACACAAAGGCAA-3′
	Reverse	5′-CTCAACTGGTGTCGTGGA-3′
RNF38	Forward	5′-TGGGAGATGACATCAAATAGGCA-3′
	Reverse	5′-GCCTGACAGGAGGACTTCTTC-3′
GAPDH	Forward	5′-ACAGTCAGCCGCATCTTCT-3′
	Reverse	5′-GACAAGCTTCCCGTTCTCAG-3′
U6	Forward	5′-CTCGCTTCGGCAGCACA-3′
	Reverse	5′-AACGCTTCACGAATTTGCGT-3′

### 2.4 Transfection assay

Small interfering RNA (siRNA) targeting circ-CFH (si-circ-CFH, sense-5′-AATGCAAAGAAAAAATTCCAT-3′; antisense-5′-AATGCAAAGAAAAAATTCCAT-3′) and its control (si-NC, sense-5′-GGCCUAAAGUAGUAGCUAUTT-3′; antisense-5′-AUAGCUACUACU UUAGGCCTT-3′) were purchased from Genechem (Shanghai, China). In addition, circ-CFH-overexpression vector (circ-CFH), RNF38-overexpression vector (RNF38), and negative control (pcDNA), miR-377-3p mimic, miR-NC, anti-miR-377-3p, and anti-miR-NC were acquired from Sangon (Shanghai, China). For transfection, Huh7 and SNU387 cells were planted into the 6-well plates (1 × 10^5^ cells/well) and cultured overnight. Then, cells were transfected with aforementioned oligonucleotides or plasmids using Lipofectamine 2000 (Applied Biosystems) according to the manufacture’s protocols.

### 2.5 3-(4, 5-Dimethylthiazol-2-yl)-2, 5-diphenyl-2H-tetrazol-3-ium bromide (MTT) assay

MTT experiments were conducted to determine the cell viability of transfected Huh7 and SNU387 cells. Briefly, Huh7 and SNU387 cells were plated in 96-well plates (3 × 10^3^ cells/well). After 48 h, 20 μL of MTT (Dojindo Laboratories, Kumamoto, Japan) was added into 96-well plates. Then, 150 μL of dimethyl sulfoxide (DMSO) was used to dissolve the formazan. The absorbance at 490 nm was recorded under an automatic microplate reader (Applied Biosystems).

### 2.6 Flow cytometry assay

Annexin V labeled with the fluorescein isothiocyanate (FITC) Apoptosis Detection Kit (Sigma-Aldrich, Louis, Missouri, USA) was used to monitor cell apoptosis. After incubation for 48 h, transfected Huh7 and SNU387 cells were harvested and then resuspended in staining buffer containing propidium iodide (PI) and FITC solution for approximately 15 min. A flow cytometer (Applied Biosystems) was used to analyze the apoptotic rate.

### 2.7 Transwell assay

The 24-well transwell chamber with polycarbonate filters (pore size, 8 μm) was used for the transwell migration assay. In brief, Huh7 and SNU387 cells (3 × 10^4^ cells) were placed in the upper chamber in 200 μL of medium without FBS (Life Technologies), while the bottom chamber was filled with 800 μL of medium supplemented with 10% FBS (Life Technologies). After 24 h of incubation, the cells migrating to the other side of the membrane were fastened with 4% formaldehyde, dyed with 0.1% crystal violet (Sigma-Aldrich), and then counted under a microscope (Olympus, Tokyo, Japan). Image J software (National Institutes of Health, Bethesda, MD, USA) was used for data analysis. The filters of the upper chamber pro-coated with Matrigel (BD Biosciences, Franklin Lakes, NJ, USA) were used for invasion assay, while other steps were consistent with the migration assay.

### 2.8 Measurement of glucose, lactate, and the ATP level

Glucose oxidase–peroxidase assay kit and lactate assay kit were purchased from Sigma-Aldrich. Transfected Huh7 and SNU387 cells were seeded into 24-well plates (1 × 10^4^ cells/well). After incubation for 48 h, the supernatant was collected and subjected to detect glucose consumption and lactate production in compliance with the manufacturer’s instructions. Furthermore, Huh7 and SNU387 cells were lysed for the measurement of the intracellular ATP level with the ATP assay kit (Sigma-Aldrich).

### 2.9 Protein extraction and western blot assay

Briefly, HCC cells or tissues were lysed in ice-cold Radio-Immunoprecipitation buffer (Sigma-Aldrich) containing 1% Triton X-100, 62.5 mM Tris-HCl (pH 6.8), and the cocktail of proteinase/phosphatase inhibitors. After the protein concentration was quantified, 50 μg of protein was seperated by 10% sodium dodecyl sulfate polyacrylamide gel electrophoresis (SDS-PAGE). Next, the wet electrophoretic transfer method was performed to transfer protein onto the nitrocellulose filter membranes (Millipore, Billerica, MA, USA). Subsequently, the membranes were probed with antibodies at 4℃, such as anti-glucose transporter type 1 (GLUT1; ab181602; 1:1,000 dilution; Abcam, Cambridge, MA, USA), anti-RNF38 (1:1,000 dilution, Boster, Wuhan, China), and anti-GAPDH (1:2,000 dilution, Boster, Wuhan, China). After overnight, the membrane was probed with goat anti-rabbit polyclonal secondary antibody (ab1500771; 3,000 dilution; Abcam) for 2 h. Then, protein signals were visualized under the Bio-Rad ChemiDoc MP imaging system (Bio-Rad).

### 2.10 Dual-luciferase reporter assay

The targets of circ-CFH and miR-377-3p were predicted by circular RNA interactome (https://circinteractome.nia.nih.gov/index.html) and TargetScanHuman (http://www.targetscan.org/vert_72/), respectively. The luciferase reporter vector was constructed by Promega (Madison, WI, USA), including WT-circ-CFH, MUT-circ-CFH, WT-RNF38 3′UTR, and MUT-RNF38 3′UTR. For the dual-luciferase reporter assay, Huh7 and SNU387 cells were seeded into 24-well plates (2 × 10^5^ cells/well) and then co-transfected with the indicated reporter vector and miR-377-3p mimic or miR-NC using Lipofectamine 2000 (Applied Biosystems). The activity of firefly luciferase was assessed with the dual-luciferase reporter assay system (Thermo Fisher Scientific, Waltham, MA, USA) and normalized to Renilla luciferase at 48 h post-transfection.

### 2.11 RNA immunoprecipitation (RIP) assay

Imprint® RNA immunoprecipitation kit was purchased from Sigma-Aldrich for RIP assay as described by Cui et al. [24]. A total of 200 μL of lysates of transfected Huh7 and SNU387 cells was incubated with magnetic beads embraced with Argonaute-2 (Ago2) or IgG at 4℃ overnight. After washing with RIP buffer, RNA complexes bound to the beads were treated with proteinase K to digest the protein. After that, the RT-qPCR assay was performed as described.

### 2.12 *In vivo* experiment

A total of 12 BALB/c nude mice (National Laboratory Animal, Center Beijing, China) were randomly assigned into two groups and fed in specific pathogen-free conditions. Huh7 cells stably transfected with sh-circ-CFH or sh-NC (Sangon) were inoculated into the right flank of nude mice (5 × 10^6^ cells/100 μL PBS/mice). Tumor formation was monitored every 4 days according to the equation: volume = 1/2 (length × width^2^). After 31 days, all mice were sacrificed and tumor tissues were removed for subsequent experiments.


**Ethical approval:** The research related to animals use has been complied with all relevant national regulations and institutional policies for the care and use of animals. All animal experiments were approved by the Animal Research Committee of Qilu Hospital, Shandong University.

### 2.13 Statistical analysis

The statistical data were analyzed using SPSS software (IBM, Chicago, IL, USA) and shown as mean ± standard deviation. The differences between two groups or multiple groups were assessed by Student’s *t*-test or one-way analysis of variance. *P* < 0.05 was considered as a significant difference. In addition, Pearson’s correlation analysis was used to assess the relationship between miR-377-3p and circ-CFH or RNF38.

## 3 Results

### 3.1 circ-CFH was overexpressed in HCC tissues and cells

**Figure 1:**
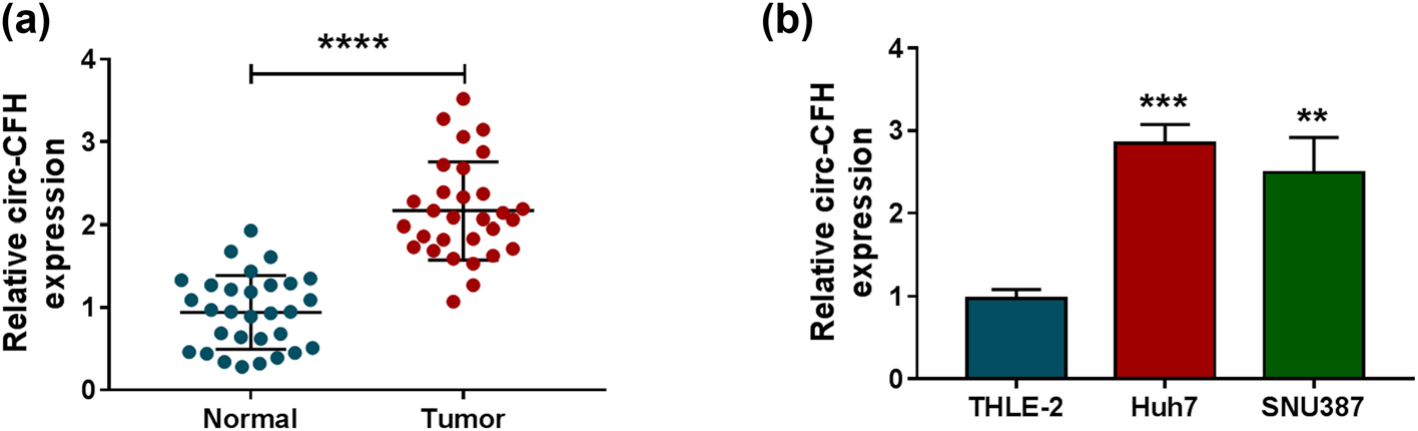
The expression level of circ-CFH in hepatocellular carcinoma tissues and cells. (a and b) The relative expression level of circ-CFH was determined by the RT-qPCR assay in hepatocellular carcinoma tissues and cells (Huh7 and SNU387), as well as in neighboring normal tissues and THLE-2 cells. **P < 0.01, ***P < 0.001, ****P < 0.0001.

The relative expression level of circ-CFH was determined by the RT-qPCR assay in HCC tissues and paired normal tissues. As presented in Figure 1a, the circ-CFH expression level was higher in HCC tissues than that in the control group. Besides, circ-CFH was more highly expressed in Huh7 and SNU387 cells than in THLE-2 cells (Figure 1b). We also investigated the correlation between circ_CFH expression and the clinical–pathological features of HCC patients. As shown in Table 1, HCC patients with high circ-CFH expression were found to have a high TNM stage (*P* = 0.028) and the presence of metastasis (*P* = 0.028). Hence, circ-CFH was overexpressed in HCC tissues and cells and might be a novel predictor for HCC prognosis.

### 3.2 Knockdown of circ-CFH inhibited proliferation, migration, invasion, and glycolysis but induced apoptosis in HCC cells

**Figure 2:**
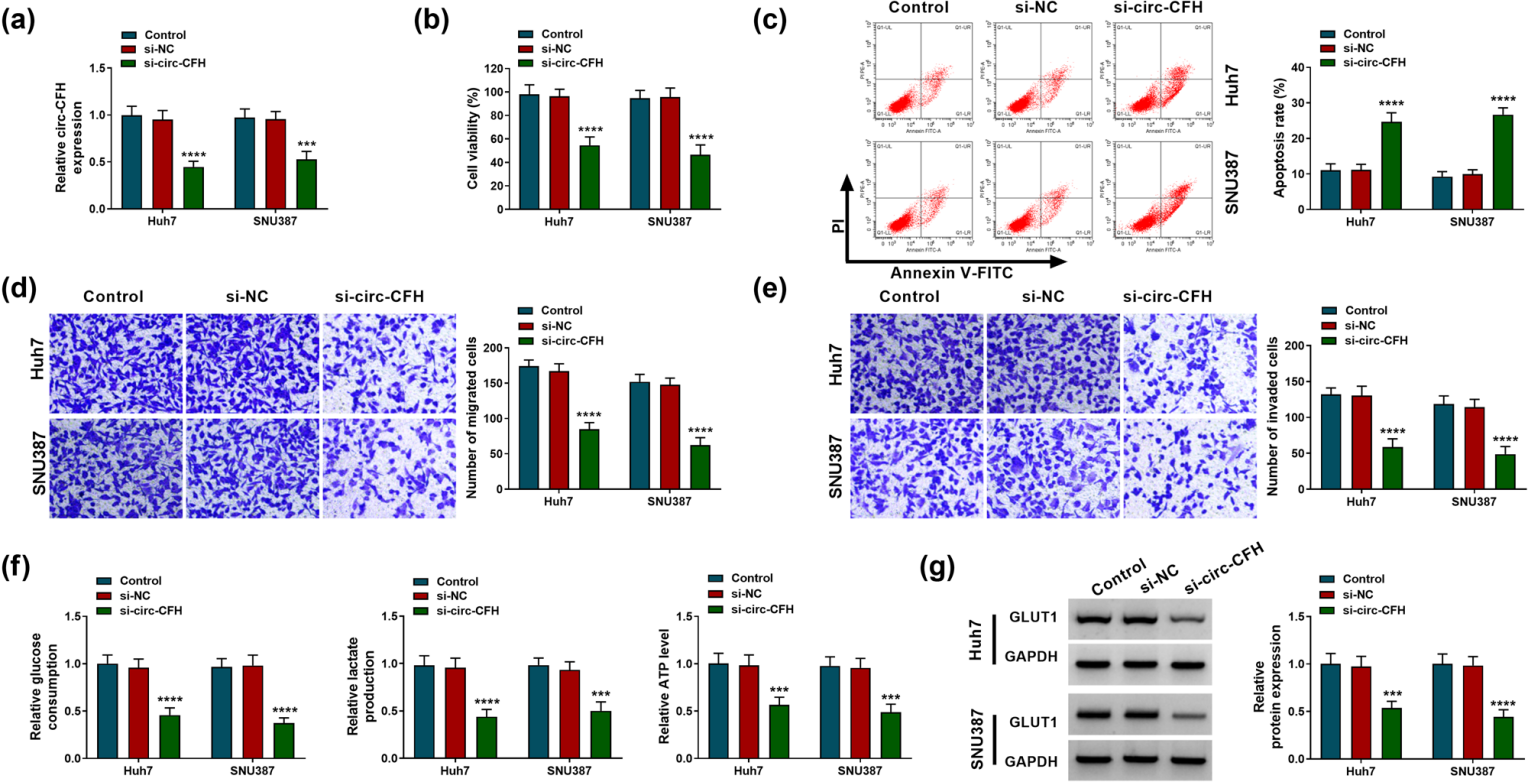
Effects of circ-CFH silencing on proliferation, apoptosis, migration, invasion, and glycolysis in hepatocellular carcinoma cells. (a–g) Huh7 and SNU387 cells were divided into three groups: control, si-NC, or si-circ-CFH. (a) The RT-qPCR assay was employed to measure the expression level of circ-CFH in Huh7 and SNU387. (b) The cell activity of Huh7 and SNU387 cells were assessed by the MTT assay. (c) The apoptotic cells were monitored by Annexin V-FITC/PI staining. (d–e) The migration and invasion of Huh7 and SNU387 cells were determined by the transwell assay. (f) The glucose consumption, lactate, and ATP level are shown in Huh7 and SNU387 cells. (g) The protein expression level of GLUT1 was quantified by western blot analysis. ***P < 0.001, ****P < 0.0001.

Given that circ-CFH was highly expressed in Huh7 and SNU387 cells, si-circ-CFH was used to knock down circ-CFH expression in HCC cells. The RT-qPCR assay suggested that the expression level of circ-CFH was inhibited in Huh7 and SNU387 cells after transfection of si-circ-CFH (Figure 2a). Besides, circ-CFH knockdown repressed the cell viability of Huh7 and SNU387 cells (Figure 2b). The flow cytometry assay suggested that the si-circ-CFH group exhibited a higher apoptosis rate than the si-NC group (Figure 2c). The Transwell assay indicated that the knockdown of circ-CFH impeded migration and invasion of HCC cells (Figure 2d and e). Glycolysis is involved in the regulation of proliferation, metastasis, immune evasion, angiogenesis, and drug resistance in HCC, which has been considered as a hallmark of liver cancer [25], as well as a strategy for HCC treatment [26]. To explore whether circ-CFH was involved in glycolysis, we measured the glucose consumption, lactate production, and ATP level in Huh7 and SNU387 cells. Knockdown of circ-CFH suppressed glucose consumption, lactate accumulation, and ATP level in Huh7 and SNU387 cells (Figure 2f). GLUT1 is a key glycolytic transporter that increases glucose uptake and catabolism via aerobic glycolysis, which is strongly associated with tumorigenesis [27,28]. Consistently, downregulation of circ-CFH decreased the protein level of GLUT1 in Huh7 and SNU387 cells compared with the si-NC group (Figure 2g). Therefore, circ-CFH knockdown could impede proliferation, migration, invasion, and glycolysis but promote apoptosis in HCC cells.

### 3.3 circ-CFH targeted miR-377-3p in HCC cells

**Figure 3:**
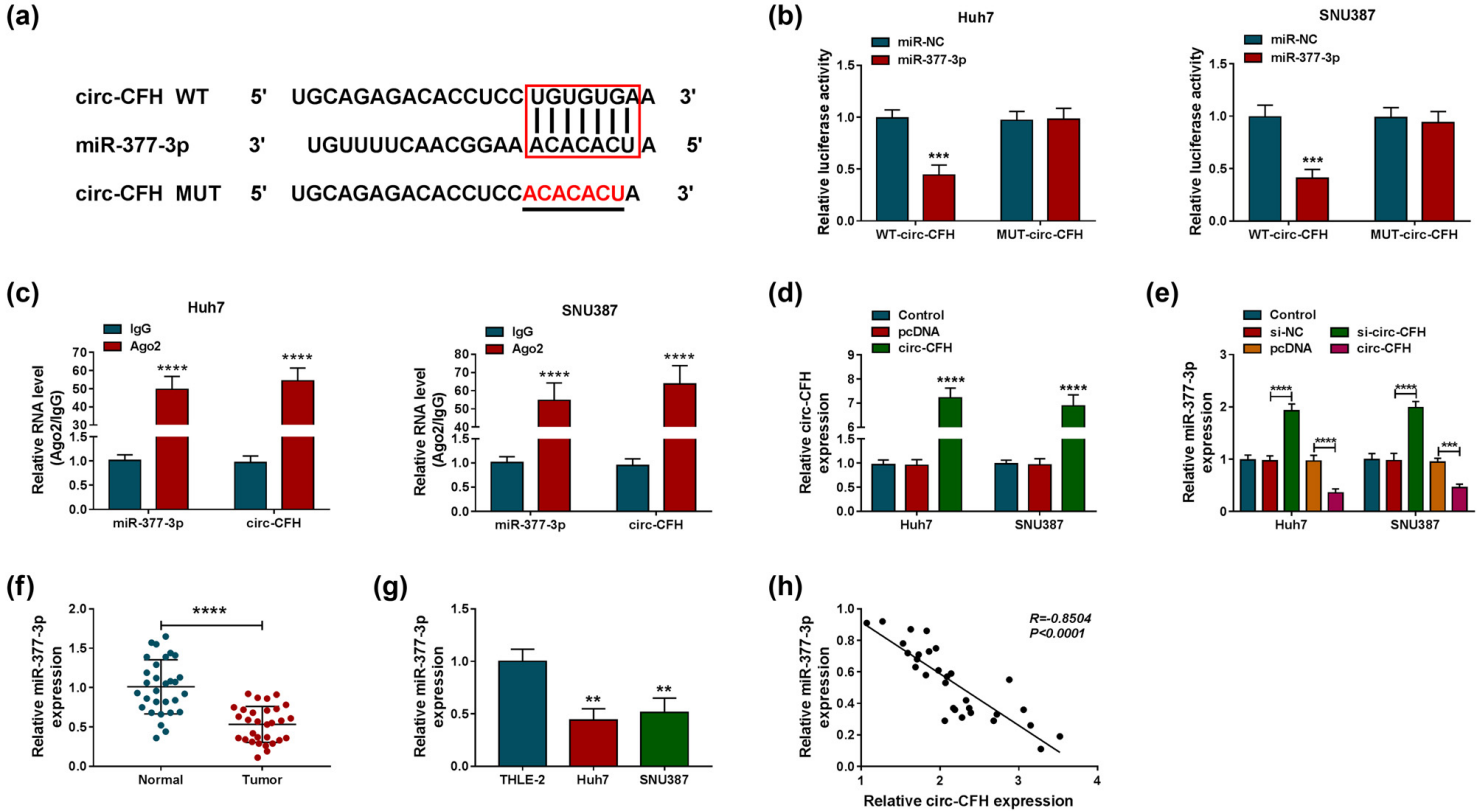
miR-377-3p was a target gene of circ-CFH. (a) The complementary sequences between miR-377-3p and circ-CFH are shown. (b and c) The interaction relationship between miR-377-3p and circ-CFH was confirmed by a dual-luciferase reporter and RIP assays. (d) The expression level of circ-CFH was estimated by RT-qPCR assay in Huh7 and SNU387 cells transfected with pcDNA or circ-CFH, with untreated cells as the control group. (e) The expression of miR-377-3p is shown by the RT-qPCR assay in Huh7 and SNU387 cells transfected with si-NC, si-circ-CFH, pcDNA, or circ-CFH, with untreated cells as the control group. (f and g) The RT-qPCR assay was performed to evaluate the miR-377-3p level in hepatocellular carcinoma tissues and cells, along with matched controls. (h) The relationship between miR-377-3p and circ-CFH was analyzed by Pearson’s correlation analysis. **P < 0.01, ***P < 0.001, ****P < 0.0001.

Bioinformatics analysis identified that miR-377-3p was a candidate target of circ-CFH, and the complementary sequence between miR-377-3p and circ-CFH is shown in Figure 3a. A dual-luciferase reporter assay was performed to determine the correlation between circ-CFH and miR-377-3p in HCC cells. Compared with the miR-NC group, overexpression of miR-377-3p significantly reduced the luciferase activity of the WT-circ-CFH group, while it did not inhibit the luciferase activity of the MUT-circ-CFH group in Huh7 and SNU387 cells (Figure 3b). In addition, circ-CFH and miR-377-3p were enriched in the Ago2 group compared with the IgG group (Figure 3c), indicating the target relationship between circ-CFH and miR-377-3p. The expression level of circ-CFH was increased in Huh7 and SNU387 cells after the transfection of circ-CFH compared with controls (Figure 3d). In addition, miR-377-3p expression was decreased in Huh7 and SNU387 cells transfected with circ-CFH overexpression vector, whereas it was enhanced in Huh7 and SNU387 cells with circ-CFH knockdown (Figure 3e). We also found that miR-377-3p expression decreased in HCC tissues and cells compared with matched controls (Figure 3f and g). Moreover, a negative correlation between miR-377-3p and circ-CFH was confirmed by Pearson’s correlation analysis in HCC tissues (Figure 3h). All data suggested that circ-CFH acted as a sponge for miR-377-3p in HCC cells.

### 3.4 circ-CFH regulated the proliferation, apoptosis, migration, invasion, and glycolysis of HCC cells by targeting miR-377-3p

**Figure 4:**
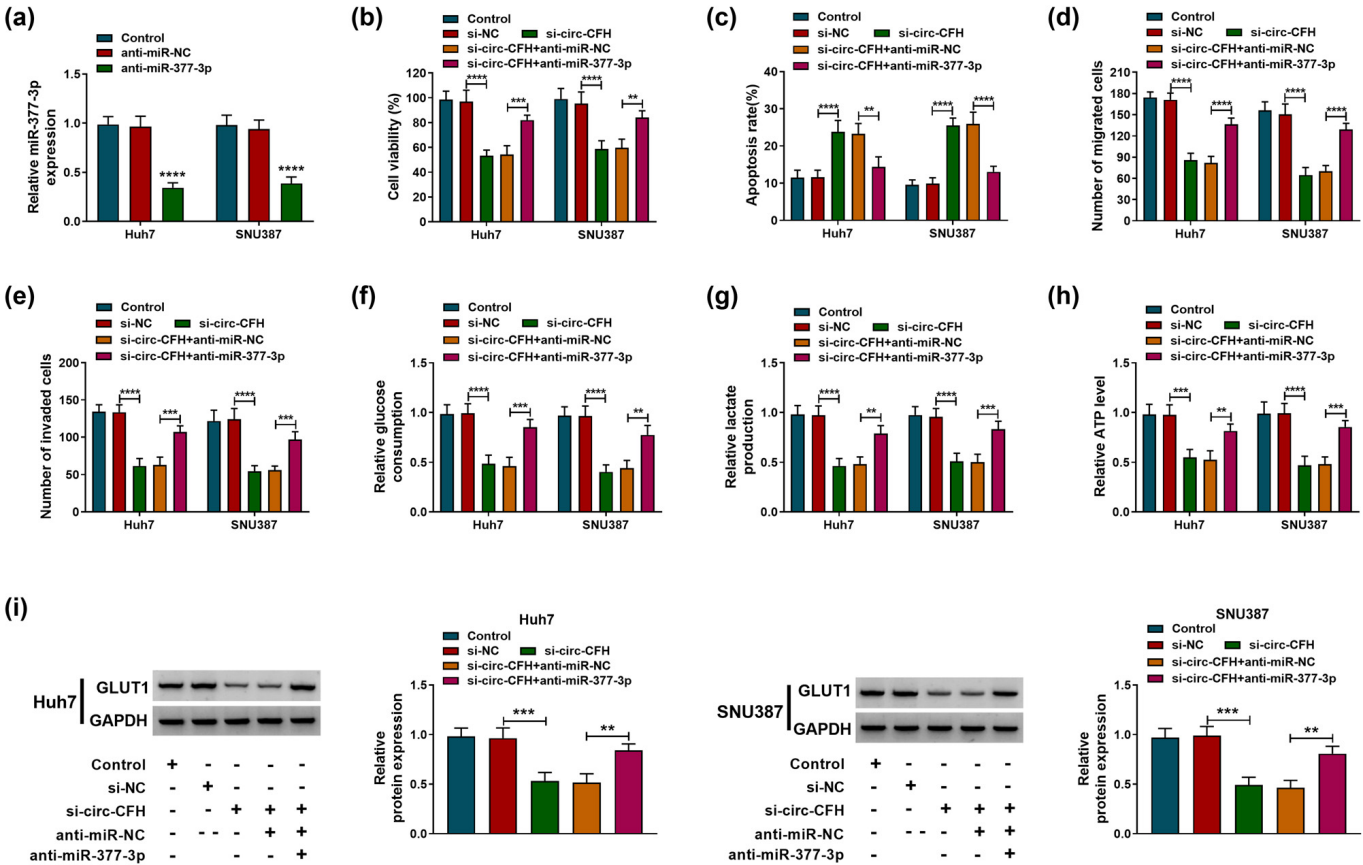
circ-CFH/miR-377-3p axis regulated proliferation, apoptosis, migration, invasion, and glycolysis of hepatocellular carcinoma cells. (a) The interference efficiency of anti-miR-377-3p was checked by the RT-qPCR assay. (b–i) Huh7 and SNU387 cells were divided into five groups: control, si-NC, si-circ-CFH, si-circ-CFH + anti-miR-NC, and si-circ-CFH + anti-miR-377-3p. (b) MTT assay was performed for examining the cell viability of Huh7 and SNU387 cells. (c) The apoptosis rate was shown by Annexin V-FITC/PI staining in Huh7 and SNU387 cells. (d and e) Transwell assay was performed in transfected Huh7 and SNU387 cells. (f–h) The glucose consumption, lactate, and ATP level of transfected Huh7 and SNU387 cells are shown. (i) Western blot analysis was used to show the expression level of GLUT1 in Huh7 and SNU387 cells. **P < 0.01, ***P < 0.001, ****P < 0.0001.

To clarify the function of the circ-CFH/miR-377-3p axis in HCC progression, Huh7 and SNU387 cells were transfected with si-NC, si-circ-CFH, si-circ-CFH + anti-miR-NC, or si-circ-CFH + anti-miR-377-3p, respectively. The RT-qPCR assay indicated that miR-377-3p was decreased in Huh7 and SNU387 cells transfected with the miR-377-3p inhibitor (Figure 4a). Besides, the decreased cell viability in the si-circ-CFH group was partly overturned by co-transfection of the miR-377-3p inhibitor (Figure 4b). Moreover, circ-CFH knockdown significantly increased apoptosis, whereas the co-transfection of anti-miR-377-3p prevented this effect (Figure 4c). The Transwell assay confirmed that the reduced cell migration and invasion abilities induced by si-circ-CFH were recovered by knockdown of miR-377-3p (Figure 4d and e). In addition, circ-CFH knockdown impeded glycolysis by inhibiting glucose consumption and decreasing lactate production and the ATP level, whereas co-transfection of anti-miR-377-3p could attenuate these effects (Figure 4f–h). Western blot analysis indicated that the protein level of GLUT1 was suppressed by si-circ-CFH, while the miR-377-3p inhibitor partly reversed the effect of si-circ-CFH on GLUT1 expression (Figure 4i). Collectively, knockdown of circ-CFH regulated proliferation, apoptosis, migration, invasion, and glycolysis in HCC cells by targeting miR-377-3p.

### 3.5 RNF38 was a target gene of miR-377-3p in HCC cells

**Figure 5:**
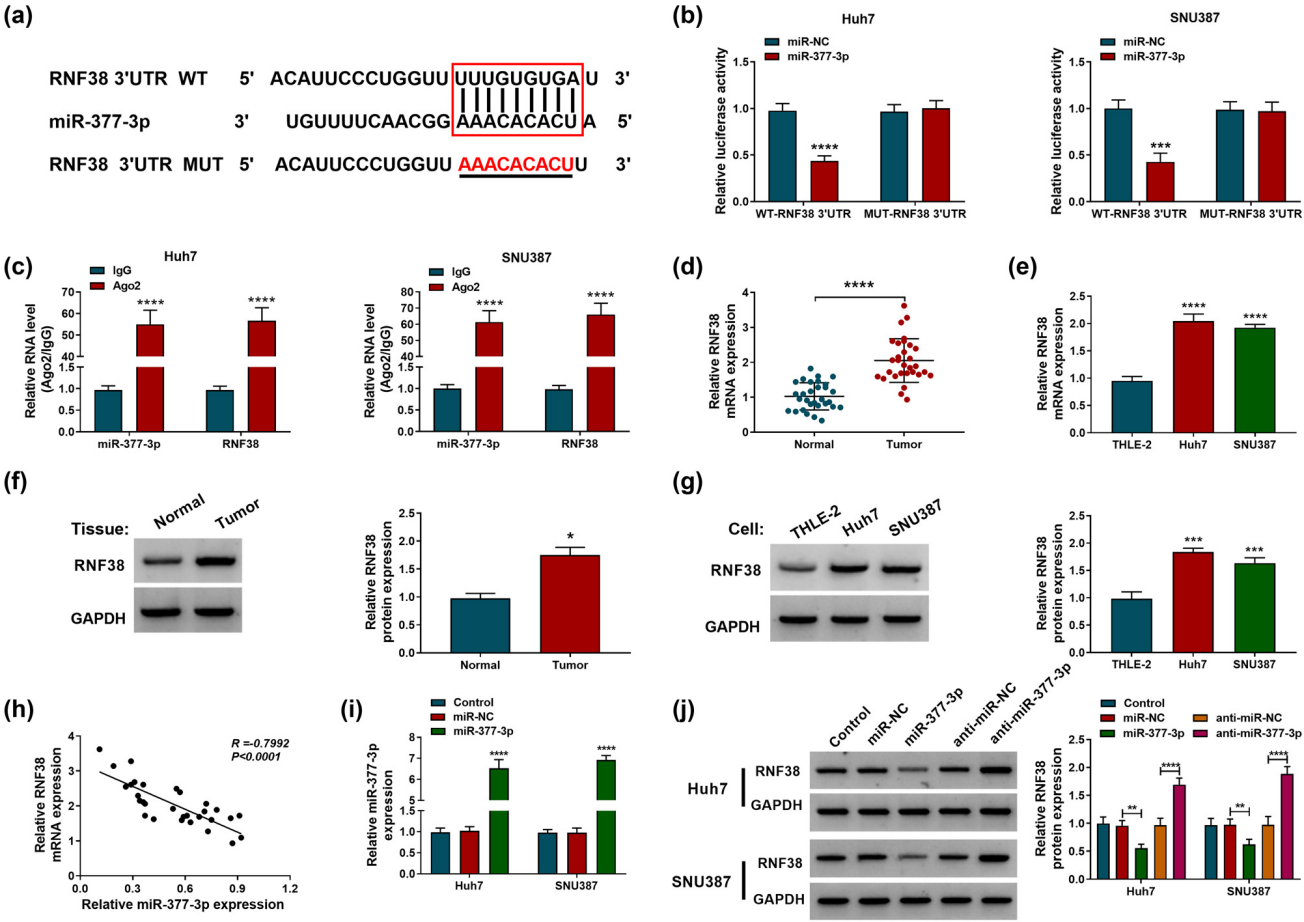
miR-377-3p regulated RNF38 expression in hepatocellular carcinoma cells. (a) The binding domain between miR-377-3p and RNF38 mRNA is shown. (b and c) Dual-luciferase reporter and RIP assays were conducted to confirm the association between miR-377-3p and RNF38 mRNA. (d–g) The mRNA and protein expression levels of RNF38 were assessed by RT-qPCR and western blot assays in hepatocellular carcinoma tissues and cells, along with matched controls. (h) Pearson’s correlation analysis was performed to analyze the relationship between miR-377-3p and RNF38 in hepatocellular carcinoma tissues. (i) The overexpression efficiency of the miR-377-3p mimic was checked by the RT-qPCR assay. (j) Western blot assay was carried out to examine the RNF38 level in Huh7 and SNU387 cells transfected with miR-NC, miR-377-3p, anti-miR-NC, or anti-miR-377-3p, with untreated cells as the control group. *P < 0.05, **P < 0.01, ***P < 0.001, ****P < 0.0001.

TargetScanHuman analysis revealed that RNF38 3′UTR sequences contained the complementary binding sites of miR-377-3p (Figure 5a). The dual-luciferase reporter assay implied that the luciferase activity of WT-RNF38 3′UTR was obviously decreased in HCC cells with miR-377-3p mimic transfection, while the luciferase activity of MUT-RNF38 3′UTR was not affected by miR-377-3p (Figure 5b). The RIP assay indicated that miR-377-3p and RNF38 were prominently enriched in the Ago2-immunoprecipitated complex in Huh7 and SNU387 cells (Figure 5c). Furthermore, the mRNA and protein expression levels of RNF38 were overexpressed in HCC tissues and cells compared with control groups (Figure 5d–g). Pearson’s correlation analysis revealed that miR-377-3p expression was negatively correlated with RNF38 expression in HCC tissues (Figure 5h). Moreover, transfection of miR-377-3p mimic increased the expression levels of miR-377-3p and RNF38 protein, while the RNF38 protein level was increased by anti-miR-377-3p transfection HCC cells (Figure 5i and j). In summary, miR-377-3p negatively regulated RNF38 expression in HCC cells.

### 3.6 MiR-377-3p regulated proliferation, apoptosis, migration, invasion, and glycolysis in HCC cells by targeting RNF38

**Figure 6:**
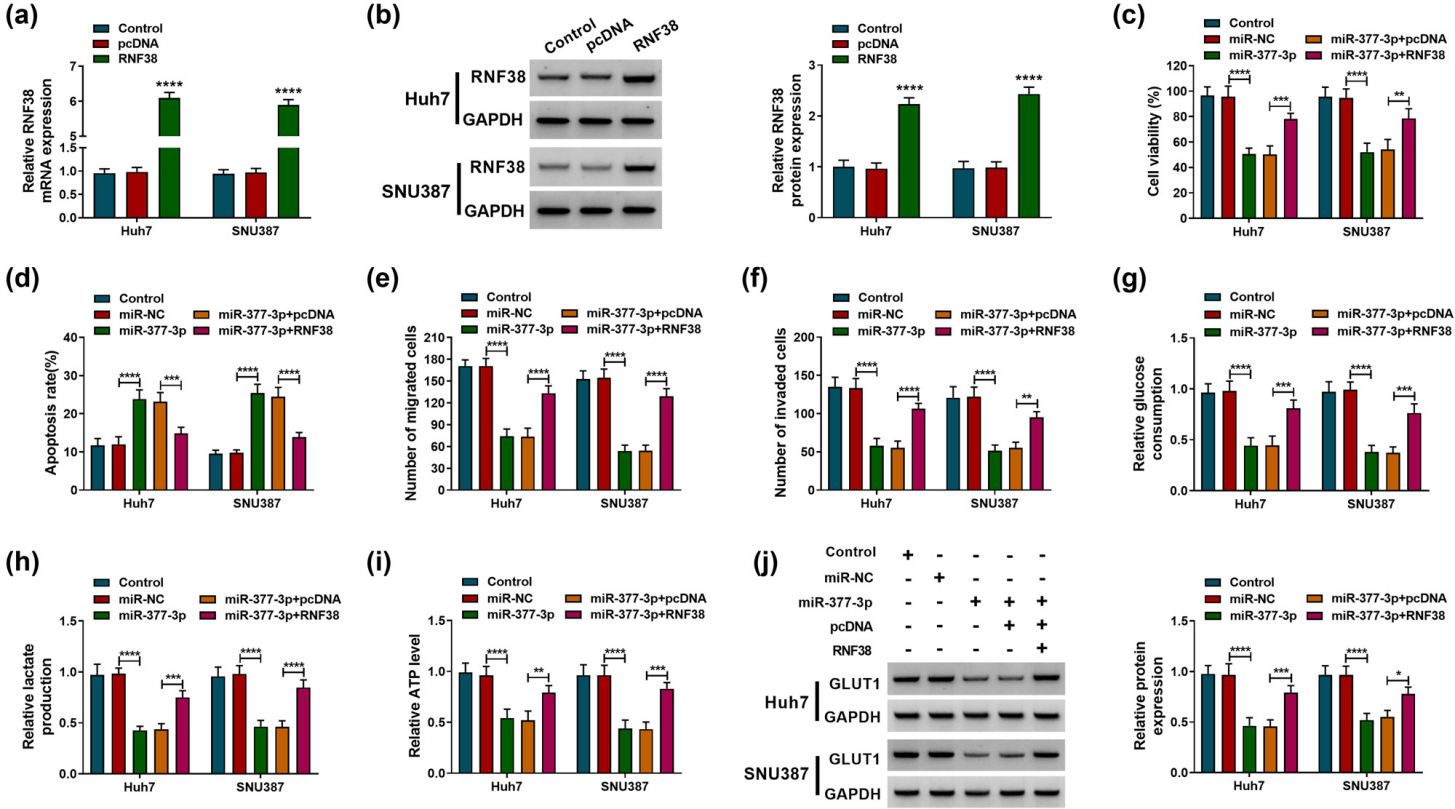
miR-377-3p regulated proliferation, apoptosis, migration, invasion, and glycolysis of hepatocellular carcinoma cells by affecting RNF38. (a and b) The overexpression efficiency of the RNF38 vector was checked by RT-qPCR and western blot analyses in Huh7 and SNU387 cells. (c–j) Huh7 and SNU387 cells were divided into five groups: control, miR-NC, miR-377-3p, miR-377-3p + pcDNA, and miR-377-3p + RNF38. (c) The cell viability of Huh7 and SNU387 cells was determined by the MTT assay. (d) Annexin V-FITC/PI staining was performed to assess the apoptosis of Huh7 and SNU387 cells. (e–f) Transwell assay was used to measure migration and invasion of Huh7 and SNU387 cells. (g–i) The glucose consumption, lactate, and ATP level are shown in Huh7 and SNU387 cells. (j) The expression level of GLUT1 was quantified by western blot analysis in Huh7 and SNU387 cells. *P < 0.05, **P < 0.01, ***P < 0.001, ****P < 0.0001.

Since RNF38 was confirmed to be a target of miR-377-3p in HCC cells, we further investigated their correlation and functions in HCC. The expression level of RNF38 was elevated in Huh7 and SNU387 cells transfected with RNF38 compared with transfection of the pcDNA vector (Figure 6a and b). The MTT assay showed that the upregulation of miR-377-3p inhibited cell growth, while this suppressive effect was weakened by RNF38 overexpression in Huh7 and SNU387 cells (Figure 6c). Overexpression of RNF38 also overturned miR-377-3p mimic-induced enhancement effect on cell apoptosis (Figure 6d). Besides, cell migration and invasion were repressed by miR-377-3p overexpression, whereas these effects were eliminated by overexpression of RNF38 (Figure 6e and f). In addition, upregulation of miR-377-3p repressed glycolysis by regulating the glucose consumption, lactate production, and ATP level, while transfection of RNF38 overturned this effect (Figure 6g and i). The downregulation of GLUT1 in Huh7 and SNU387 cells that was induced by the miR-377-3p mimic was also abolished by RNF38 overexpression (Figure 6j). Hence, overexpression of miR-377-3p repressed proliferation, migration, invasion, and glycolysis while it induced apoptosis in HCC cells by targeting RNF38.

### 3.7 circ-CFH regulated RNF38 expression by targeting miR-377-3p

**Figure 7:**
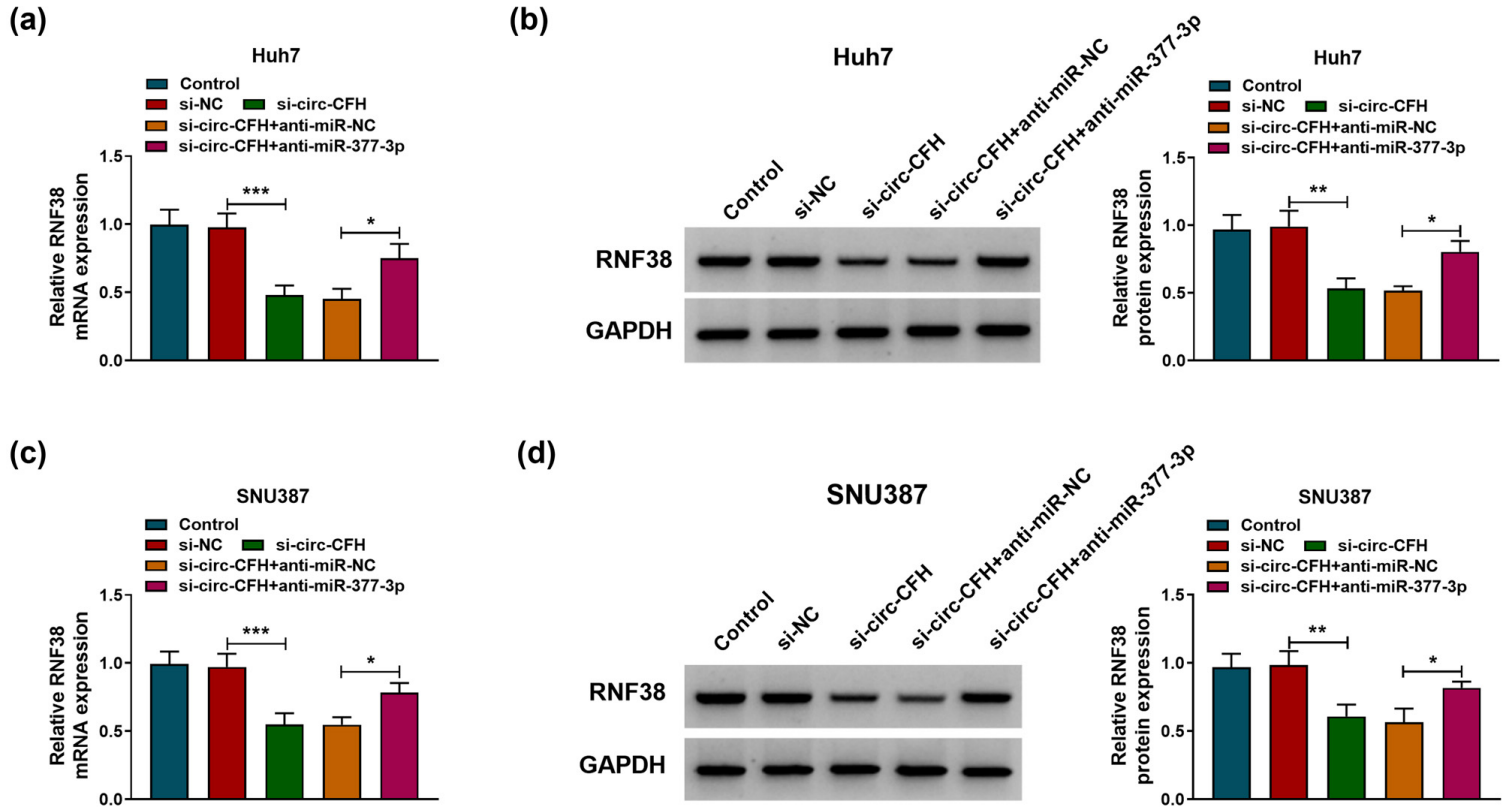
circ-CFH/miR-377-3p/RNF38 axis in hepatocellular carcinoma cells. (a–d) The mRNA and protein expression levels of RNF38 are shown by RT-qPCR and western blot assays in Huh7 and SNU387 cells transfected with si-NC, si-circ-CFH, si-circ-CFH + anti-miR-NC, or si-circ-CFH + anti-miR-377-3p, with untreated cells as the control group. *P < 0.05. **P < 0.01, ***P < 0.001.

To explore whether circ-CFH regulated RNF38 expression by sponging miR-377-3p, Huh7, and SNU387 cells were transfected with si-NC, si-circ-CFH, si-circ-CFH + anti-miR-NC, or si-circ-CFH + anti-miR-377-3p, respectively. As presented in Figure 7a–d, co-transfection of anti-miR-377-3p partly abolished the suppression effects of si-circ-CFH on RNF38 mRNA and protein levels in Huh7 and SNU387 cells. Therefore, circ-CFH regulated the expression of RNF38 by sponging miR-377-3p in HCC.

### 3.8 Silencing of circ-CFH inhibited tumor growth *in vivo*

**Figure 8:**
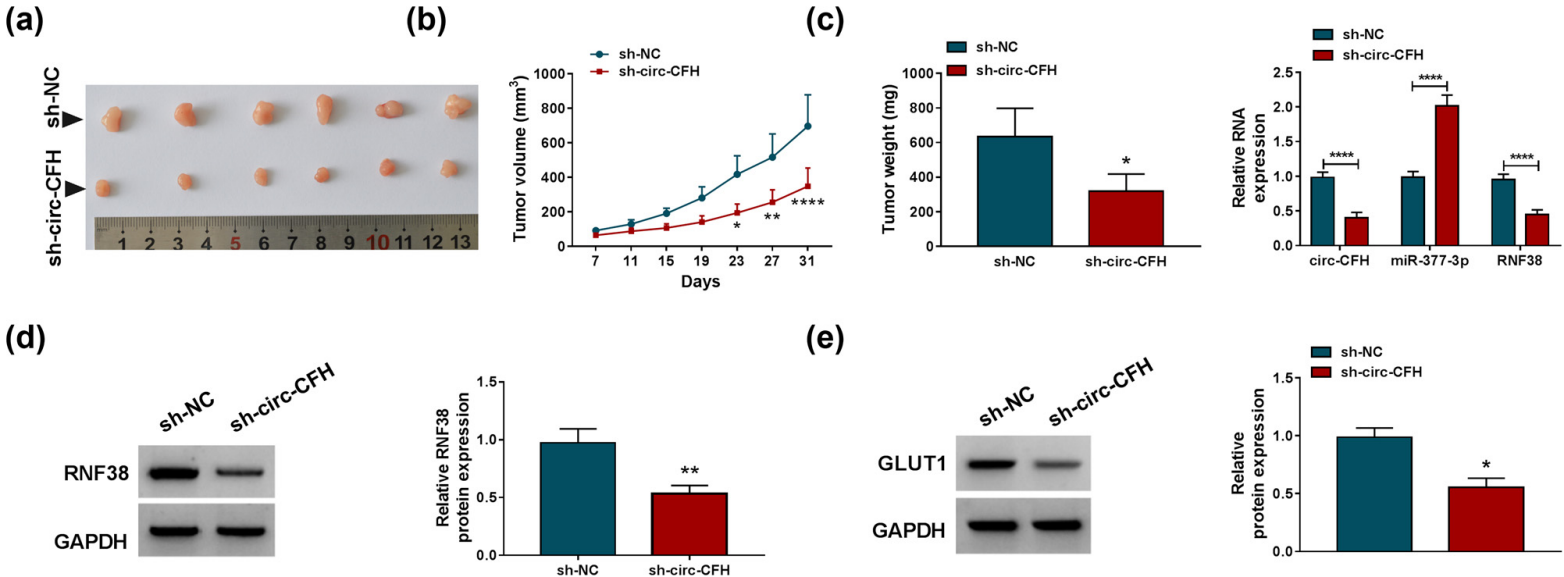
Silencing of circ-CFH repressed tumor growth *in vivo*. (a and b) The growth curves and weight of xenograft tumors are shown. (c) The expression levels of circ-CFH, miR-377-3p, and RNF38 were estimated with the RT-qPCR assay in dissected tumor tissues from different groups. (d and e) Western blot assay was conducted to quantify protein levels of RNF38 and GLUT1. *P < 0.05. **P < 0.01, ****P < 0.0001.

A xenograft tumor model was established to uncover the function of circ-CFH *in vivo*. As shown in Figure 8a–c, the tumor volume and weight of the sh-circ-CFH group were significantly reduced compared with the sh-NC group. Besides, the silencing of circ-CFH inhibited RNF38 and circ-CFH expression while it enhanced miR-377-3p expression in xenograft tumor tissues (Figure 8c). Western blot assay indicated that RNF38 and GLUT1 protein levels were lower in the sh-circ-CFH group than that in the sh-NC group (Figure 8d–e). Therefore, we speculated that circ-CFH regulated the progression of HCC by the miR-377-3p/RNF38 axis *in vivo*.

## 4 Discussion

In this research, we investigated the possible molecular mechanism of circ-CFH in the HCC process. The results suggested that circ-CFH was upregulated in HCC tissues and cells. Mechanistically, circ-CFH enhanced the malignant progression of HCC by acting as a miR-377-3p sponge to increase RNF38 expression in HCC cells.

With the development of high-throughput sequencing and novel computational approaches, more and more circRNAs have been recognized and were found to exert crucial roles in human diseases and cancers [29,30]. In contrast with linear RNAs, circRNAs are more stable and exhibit tissue- and stage-specific expression, making them possible therapeutic candidates [29,30]. Several circRNAs have been perceived as potential diagnostic and therapeutic targets for HCC. For instance, circ_104075 was found to have the potential to act as a diagnostic biomarker for HCC since it enhanced the tumorigenesis of HCC and had a prominent diagnostic performance [31]. Besides, Yu et al. uncovered that hsa_circ_0001445 inhibited the growth and migration of HCC *in vitro* and *in vivo*, which could be a potential therapeutic target for HCC [32]. Consistent with previous research, we focused on investigating the function of circ-CFH in HCC. In the present research, circ-CFH was highly expressed in HCC tissues and cells, and its high expression was closely related to tumor metastasis and the TNM stage. Notably, tumor cells tend to acquire energy and macromolecular intermediates through glycolysis even under sufficient oxygen conditions, which is closely associated with resistance to current therapy and poor prognosis patients [33,34]. Therefore, cell proliferation, migration, invasion, apoptosis, and glycolysis were investigated in HCC cells. In the present research, knockdown of circ-CFH reduced cell viability, migration, invasion, and glycolysis but promoted apoptosis in HCC cells *in vitro*. The silencing of circ-CFH also inhibited tumor growth *in vivo*.

Additionally, the circRNA-mediated ceRNA network has been widely recognized on account of their important roles in human malignant tumors [35]. circ-CFH could function as a miR-1250-3p sponge to regulate cell viability, proliferation, and invasion of hepatoblastoma cells *in vitro* [36]. Liu et al. revealed that the downregulation of circ-CFH repressed cell viability, proliferation, and invasion in HCC cells by targeting miR-7 [12]. In the current research, miR-377-3p was predicted to be a target of circ-CFH in HCC. Hence, we further investigated the contributions of circ-CFH in HCC with a specific focus on its association relationship with miR-377-3p. Chen et al. suggested that the cell growth and invasion of HCC cells were suppressed by overexpression of miR-377 [37]. In our research, miR-377-3p was lowly expressed in HCC tissues and cells and was negatively correlated with the circ-CFH expression level in HCC tissues. Besides, miR-377-3p downregulation partly overturned the suppression effect of circ-CFH knockdown on HCC progression. Hence, circ-CFH acted as a sponge for miR-377-3p to regulate tumorigenesis of HCC.

It is widely known that miRNA exerted its function in cancers by inducing mRNA degradation or translation inhibition of target mRNAs [13]. Ge et al. reported that miR-377 could target 3′UTR of B-cell lymphoma-extra large (Bcl-xL) to reduce cell activity and enhance apoptosis in HCC [38]. Furthermore, miR-377-3p was negatively regulated by hsa_circ_103809 and regulated the expression of FGFR1, thereby inhibiting the progression of HCC [18]. Our data implied that RNF38 harbored the complementary binding sites of miR-377-3p and was confirmed to be negatively regulated by miR-377-3p. RNF38 has been suggested to play an important role in cancers. Overexpression of RNF38 could induce p53 ubiquitination by serving as a functional ubiquitin-protein ligase to participate in the regulation of tumorigenesis [39,40]. Additionally, Peng et al. revealed that RNF38 induced the EMT process of HCC cells by ubiquitinating and degrading neuroblast differentiation-associated protein (AHNAK, an inhibitor of TGF-β signaling) [41]. Herein, RNF38 was found to be highly expressed in HCC tissues and was negatively correlated with miR-377-3p expression in HCC. In agreement with the previous study [42], our data indicated that RNF38 promoted the HCC process by accelerating proliferation, migration, and invasion and inhibiting apoptosis in HCC cells. Besides, we found that miR-377-3p functioned as a carcinoma inhibitor by inhibiting cell activity and mobility and stimulating apoptosis of HCC cells via regulating RNF38. In addition, it was found that circ-CFH could regulate the expression of RNF38 by sponging miR-377-3p in HCC. Conclusively, our data illuminated that circ-CFH exerted the oncogenic function in HCC by acting as a miR-377-3p sponge to regulate RNF38 expression.

In summary, circ-CFH played an important role in the tumorigenesis of HCC. The silencing of circ-CFH impeded HCC progression by regulating proliferation, apoptosis, migration, invasion, and glycolysis in HCC cells. Mechanistically, circ-CFH exerted its biological roles in HCC by the miR-377-3p/RNF38 axis, which provided theoretical support for the development of therapeutic strategies for HCC.
